# Pecan (*Carya illinoinensis*) Shells as a Source of Antioxidants: Implications for Oxidative Stress-Driven Pathologies

**DOI:** 10.3390/molecules31060993

**Published:** 2026-03-16

**Authors:** Ifeoma Roseline Ezeanolue, Judith George, Precious Aimalohi Ohioze, Oluwapelumi Oloyede Oyeniyi, Jasper Okoro Godwin Elechi, Monica Rosa Loizzo, Pierluigi Plastina

**Affiliations:** 1 Department of Pharmacy and Health and Nutritional Sciences, University of Calabria, 87036 Arcavacata di Rende, Italy; ezeanolueifeoma@gmail.com; 2 Department of Plant Biology and Biotechnology, Faculty of Life Sciences, University of Benin, Benin City 300283, Nigeria; judith.george@lifesci.uniben.edu; 3 Edo State Traditional Medicine Board, Benin City 300283, Nigeria; 4 Edo State Hospital Management Agency, Benin City 300241, Nigeria; precious.ohioze@lifesci.uniben.edu; 5 School of Pharmaceutical Sciences of Ribeirão Preto, University of São Paulo, Ribeirão Preto 14040-903, SP, Brazil; pelu.oyeniyi@usp.br; 6 Department of Food and Human Nutritional Sciences, University of Manitoba, Winnipeg, MB R3T 2N2, Canada; jasper.elechi@ufpe.br; 7 St. Boniface Hospital Albrechtsen Research Centre, Winnipeg, MB R2H 2A6, Canada

**Keywords:** pecan nutshell, oxidative stress, antioxidants, chronic disease, bioavailability

## Abstract

Pecan nutshells (PNS), once considered agricultural waste, are now recognized as a sustainable source of natural antioxidants with potential therapeutic benefits against oxidative stress-related diseases. This narrative review synthesized evidence from the last decade, including predominantly in vitro and in vivo studies, with limited clinical evidence. PNS are particularly rich in polyphenols (gallic acid, ellagic acid, vanillic acid, catechins), with phenolic and flavonoid concentrations reported to be 5–20 times higher than those in the edible kernels. Their antioxidant actions involve free radical scavenging, metal chelation, enhancement of enzymatic defenses, and modulation of redox signalling. Preclinical findings suggest protective roles in cardiovascular disease, diabetes, neurodegeneration, and cancer, mediated through reduced lipid peroxidation, improved glucose metabolism, neuroprotection, and anticarcinogenic activity. However, variability in extraction methods, cultivar differences, and bioavailability issues remain major challenges. Standardized clinical studies are needed to validate the therapeutic potential of PNS as a sustainable antioxidant source.

## 1. Introduction

The rapid growth of the global population has led to a corresponding increase in agricultural waste generation [1]. This waste originates from diverse sources such as crop residues, agro-industrial processes, livestock production, and aquaculture [1]. Agro-industrial activities contribute significantly, producing by-products like husks, shells, pomace, molasses, skins, and bagasse [2,3]. Inadequate management of such waste not only threatens sustainable farming but also poses risks to food security, public health, and the environment. For instance, poor disposal practices contribute to greenhouse gas emissions, thereby exacerbating global warming [2,4]. According to the Food and Agriculture Organization (FAO), millions of tons of agro-waste are produced annually worldwide, underlining the urgent need for innovative waste management strategies [5]. To address these challenges, upcycling and valorization strategies have been proposed as sustainable solutions. Among these, the recovery of bioactive compounds has gained considerable attention, as it enables the conversion of waste into valuable resources for food, pharmaceutical, cosmetic, and other industries [3,4]. Beyond mitigating environmental hazards, by-products also contain vitamins, minerals, phytochemicals, fiber, proteins, and oils. These compounds provide health-promoting effects, including antioxidant, antimicrobial, anti-aging, moisturizing, and regenerative benefits, highlighting the dual value of waste valorization [6].

Plant foods like nuts are not only a rich source of essential nutrients but also packed with phytochemicals and antioxidants that help the body fight oxidative stress and inflammation. Commonly consumed varieties include almonds, Brazil nuts, cashews, hazelnuts, macadamias, peanuts (though technically a legume), pecans, pine nuts, pistachios, and walnuts. Beyond being enjoyed as snacks, nuts serve as valuable raw materials in food industry, where they are used in confectionery, baked products, beverages, and cooking oils. Due to their strong antioxidant and anti-inflammatory properties, regular nut consumption has been linked to better cardiometabolic health. Specifically, studies have shown inverse associations between nut consumption and all-cause mortality, reinforcing their role as functional foods in public health nutrition [7,8,9].

The pecan nut (*Carya illinoinensis* (Wangenh.) K. Koch) is a monoecious, heterodichogamous, deciduous nut tree native to the southern United States, with its cultivation now extending to South America, Africa, and parts of Asia due to favorable climates and high global demand. It is a member of the *Juglandaceae* family and can grow up to 60 m in height and live for a very long time. It is classified as a fruit tree (angiosperm), whose fruit is best classified as a drupe, pseudodrupe, or a drupaceous nut rather than a proper nut [10]. Pecan kernels are particularly notable for their nutritional density, containing approximately 72% fat (predominantly monounsaturated oleic acid), 9% protein, 14% carbohydrates, and 9.6% dietary fiber. They are rich in essential micronutrients including vitamin E (tocopherols), thiamine, magnesium, zinc, manganese, and copper. The favorable fatty acid profile, combined with high polyphenol content (including ellagic acid, catechins, and proanthocyanidins), contributes to their cardioprotective effects. Epidemiological studies have shown that regular pecan consumption (approximately 42–68 g/day) is associated with improved lipid profiles, reduced LDL oxidation, enhanced endothelial function, and better glycemic control. These health benefits have driven increased global demand for pecan kernels, simultaneously generating substantial quantities of shell waste that warrant valorization [11,12].

**Figure 1:**
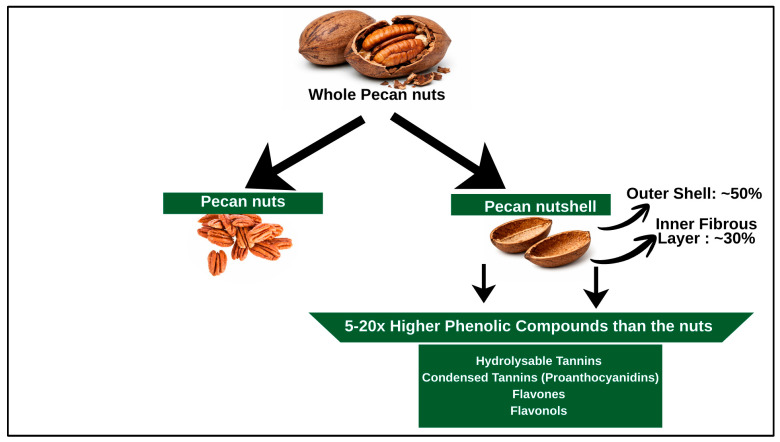
Simple pictorial representation of the parts of the Pecan nut, with the main focus on the shell.

Pecan processing generates a high proportion of shells (the hard outer covering of the kernel). The leaves of the tree are used as tea for the treatment of digestive, inflammatory processes and skin problems, while the infusion of its rinds is used for chronic diarrhea or as a tonic in anemia [11,13]. Regarding the shells specifically, historical records indicate limited traditional medicinal use, though Native American communities in the southern United States reportedly used pecan shell decoctions as astringents for skin conditions and diarrhea, likely due to their high tannin content. More commonly, pecan shells have been utilized for non-medicinal purposes including natural dyeing (producing brown/tan colors), livestock bedding, smoking meats, and fuel. The lack of documented traditional medicinal applications for pecan shells, in contrast to leaves and bark, may partially explain the delayed scientific interest in their therapeutic potential [14,15]. Pecan nutshells (PNS) are the most abundant and valuable byproduct which represents 40–50% waste from the processing of pecans. It comprises cellulose (fibrous homopolysaccharide made up entirely from β-(1–4)-linked glucose units), hemicellulose (amorphous heteropolysaccharide composed of many different sugar monomers), lignin (a non-carbohydrate polymer synthesized from the polymerization of phenolic alcohols, which produce a heterogeneous and polydisperse polymer), and other carbohydrates, also known as insoluble fiber [16]. Figure 1 provides a simple pictorial representation of the parts of the pecan nut, with emphasis on the shell, which is increasingly recognized as a source of valuable bioactive compounds.

Among the diverse array of tree nuts, pecans are selected for this review for several compelling reasons. First, pecan production generates substantial waste streams, where shells constitute 40–50% of total nut weight, representing approximately 150,000 tons of shell waste annually from global production of ~300,000 tons (2022 data). Second, unlike many other nutshells that have been extensively studied (e.g., almond, walnut), pecan shells remain comparatively underexplored despite preliminary evidence of exceptionally high phenolic content (5–20 times higher than kernels). Third, the unique phytochemical profile of pecan shells, particularly their abundance of ellagic acid derivatives, condensed tannins, and hydrolyzable tannins, distinguishes them from other nut by-products and suggests distinct therapeutic potential. Fourth, the increasing global cultivation of pecans in the United States, Mexico, South Africa, Australia, and emerging regions creates both an environmental challenge (waste management) and an economic opportunity (value-added products). Finally, from a circular economy perspective, valorizing pecan shells aligns with UN Sustainable Development Goals while potentially providing low-cost antioxidant ingredients for food, pharmaceutical, and cosmetic applications. These factors collectively justify focused investigation of pecan shells as a priority agricultural by-product for bioactive compound recovery.

In recent years, PNS have attracted considerable scientific interest because they contain higher concentrations of phenolic compounds and flavonoids than the kernels themselves. The processing of PNS generates various value-added products, including extracts obtained using different solvents, nutshell flour, and lignocellulosic biomass. The PNS biomass is a natural resource of lignocellulosic materials, which can be successfully used as fillers in polymer matrices [3]. In addition, another sustainable approach to utilize PNS is in the production of activated carbons for supercapacitors [17]. These non-food applications highlight the versatility of PNS in both health-related and industrial contexts, aligning with the United Nations Sustainable Development Goals (SDGs). For instance, their role in “Zero Hunger” (SDG 2) and “Responsible Consumption and Production” (SDG 12) reflects their importance in sustainable agriculture and waste reduction [18]. Although it is evident that PNS’s major phenolics are gallic acids, chlorogenic acids and catechins, these compounds have poor bioavailability because of low solubility, hence the need for encapsulation to improve the bioavailability of these compounds in PNS [19]. Extracts from PNS through hydroalcoholic extraction have been found to be used in the development of active packaging materials to enhance the shelf life and quality of food products [20]. Based on the effect on health, some studies have reported the health benefits of the extract from PNS such as its potential in the treatment of oxidative stress-related disorders like diabetes and hypercholesterolemia [21].

Despite this promising preliminary evidence, comprehensive systematic studies on the therapeutic potential of PNS have been notably absent from literature until recently. Several factors explain this research gap. First, agricultural by-products have historically been viewed as waste rather than valuable resources, with research funding preferentially directed toward edible kernel components. Second, the complex and variable nature of shell composition influenced by cultivar, growing conditions, harvest timing, and storage presents significant standardization challenges that have deterred pharmaceutical-grade development. Third, bioavailability concerns related to high-molecular-weight tannins and poor aqueous solubility of key phenolics have raised questions about oral efficacy, requiring advanced formulation strategies. Fourth, the lack of validated extraction protocols and phytochemical reference standards has hindered comparative studies and reproducibility. Fifth, regulatory pathways for agricultural waste-derived ingredients remain unclear in many jurisdictions, creating commercialization uncertainty. Finally, the relatively recent emergence of “waste valorization” and “circular economy” concepts (post-2015) has only now created the scientific and economic momentum needed for systematic investigation. This review addresses this critical gap by synthesizing the available evidence and identifying priority research directions for translating PNS from agricultural waste to therapeutic resource.

Oxidative stress itself is a key concept in understanding these therapeutic applications. It occurs when there is an excessive buildup of unstable molecules, such as oxygen- and nitrogen-derived free radicals, in human cells. These molecules are essential for maintaining health because they play key roles in defense against infections and the regulation of biological processes. However, overproduction can disrupt cellular balance and lead to several health conditions, including kidney disease, diabetes, and neurological disorders [22]. Normally, the body maintains a balance by removing excess free radicals. When this equilibrium is lost, oxidative stress sets in, causing damage to proteins, lipids, and DNA. One consequence of this imbalance is the formation of advanced glycation end products (glycotoxins), which alter the structure of proteins and DNA, thereby contributing to chronic degenerative diseases, ageing, and acute conditions such as trauma and stroke [23].

Given its central role in disease pathology, oxidative stress has become a major focus of preventive and therapeutic research. To combat oxidative stress, the body relies on antioxidant mechanisms that convert harmful radicals into harmless molecules, bind and inactivate heavy metals, interrupt free radical chain reactions, and repair damaged molecules [24]. However, when these natural defenses are overwhelmed, external support becomes necessary. Antioxidant therapy, which involves using natural or synthetic substances to neutralize free radicals, has been explored as a strategy to restore redox balance [25]. Such therapies have proven useful in various disease conditions, including hypertension [26]. Natural antioxidants derived mainly from plant-based foods are particularly valued because of their safety, affordability, and availability. They include polyphenols, flavonoids, anthocyanins, vitamins, and minerals, all of which help reduce oxidative damage [14]. Dietary studies show that individuals whose diets are rich in nuts and other plant-based foods have a lower prevalence of long-term degenerative diseases due to the protective effects of natural antioxidants [27]. For example, recent trials suggest that higher nut intake is associated not only with cardiometabolic benefits but also with improved cognitive outcomes, linking nut bioactives to brain health. Beyond conventional dietary sources, increasing attention has turned to agricultural by-products as sustainable reservoirs of antioxidant compounds [12]. This shift not only addresses the global need for affordable therapeutic strategies but also supports waste reduction and value addition in food systems. Despite these promising findings, PNS remain underutilized compared to other nut by-products. While some trials have shown that PNS can play an important role as a therapeutic agent, no comprehensive report has been made on the effect of PNS on these stated diseases. This paper aims to explore the antioxidant potential of PNS and its possible therapeutic applications in preventing or mitigating oxidative stress-related diseases.

## 2. Literature Search Methodology

This narrative review was conducted by systematically searching the published literature on pecan nutshell (PNS) and its potential therapeutic roles in oxidative stress–related diseases. This comprehensive review synthesizes and critically appraises the current published literature, focusing on existing evidence rather than reporting novel experimental findings.

This approach is essential for identifying knowledge gaps, establishing research priorities, and providing a foundation for future experimental investigations. The narrative review format was chosen to accommodate the diverse methodologies, model systems, and outcome measures reported across the PNS literature, which preclude formal meta-analysis. Relevant studies were retrieved from PubMed, Scopus, Web of Science, and Google Scholar, using combinations of the keywords: “pecan nutshell,” “pecan nutshell extract,” “antioxidant,” “oxidative stress,” “bioavailability,” and “clinical application.” Additional search terms included: “*Carya illinoinensis* shell,” “pecan shell polyphenols,” “pecan waste valorization,” “ellagic acid,” “proanthocyanidins,” and disease-specific terms (cardiovascular, diabetes, neurodegeneration, cancer) combined with “pecan shell” using Boolean operators. The search covered articles published in English between 2015 and 2025. This timeframe was selected to capture recent advances in extraction technologies, analytical methods, and mechanistic understanding, while acknowledging that foundational earlier studies (pre-2015) were included when they provided critical context or represented landmark findings not superseded by recent work. Both preclinical (in vitro and animal) and clinical studies were included, alongside review articles that provided mechanistic insights. Boolean operators (AND, OR) and wildcards (*, $, among others) were also used to get more accurate results.

The initial database search identified approximately 450 records. After screening titles and abstracts, 180 articles were deemed potentially eligible and underwent full-text assessment. Reference lists of the included studies were manually screened to capture additional relevant publications (snowball sampling). Grey literature, conference abstracts, and unpublished data were excluded to preserve methodological rigor and reproducibility.

Studies were included if they addressed at least one of the following: Antioxidant properties of PNS; Phytochemical composition of PNS, particularly polyphenols; Bioavailability and metabolism of active compounds; Potential therapeutic applications in oxidative stress–related conditions. Additionally, studies reporting extraction methodologies, safety/toxicity data, or comparative analyses with other nut by-products were included. Both positive and negative findings were eligible for inclusion to avoid publication bias. Studies were excluded if they were unrelated to oxidative stress or antioxidant activity, or if they lacked relevance to human health outcomes or preclinical disease models. Specifically, excluded studies included: studies focusing solely on non-health applications (e.g., biofuel, biochar, industrial materials without bioactivity data); studies on pecan kernels without shell data; animal studies without clear oxidative stress or disease endpoints; and studies with insufficient methodological detail to assess validity. Articles related to hyperglycemia, endothelial dysfunction, neurodegenerative diseases, and cancer were selected and grouped. Data from the selected studies were synthesized narratively, with findings organized into major themes: oxidative stress and chronic disease links, antioxidant therapy and natural sources, phytochemical composition of PNS, and their therapeutic applications. For each disease category, evidence was stratified by model system (in vitro cell culture, ex vivo tissue, in vivo animal models, human studies) to facilitate critical appraisal of translational relevance. Where multiple studies addressed the same question, findings were synthesized to identify consensus, contradictions, and methodological factors influencing outcomes. Quality assessment considered sample size, control groups, dose–response evaluation, mechanistic investigation, and reproducibility across independent laboratories.

## 3. Results

### 3.1. Keywords Clustering

**Figure 2:**
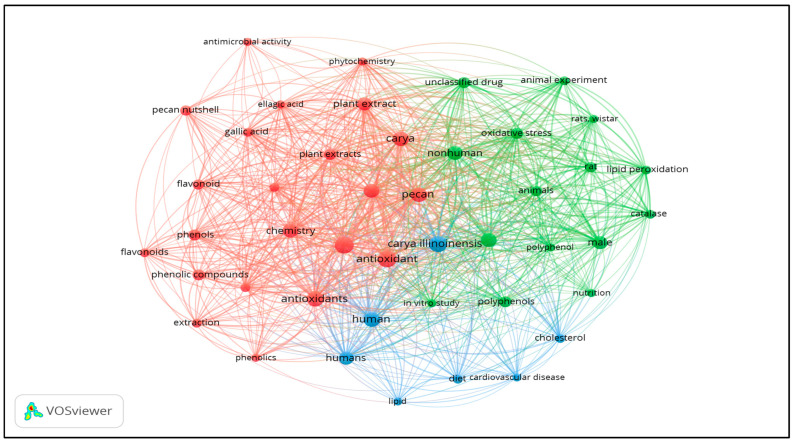
Network of keywords frequently co-occurring in Scopus databases regarding Pecan nutshell and oxidative stress disorders. Three distinct clusters emerged: RED = extraction and phytochemical characterization; GREEN = preclinical (in vitro/in vivo) mechanistic studies; BLUE = human clinical and dietary studies. Node size reflects keyword frequency; connecting lines indicate co-occurrence strength. Analysis based on 85 Scopus-indexed publications with ≥5 keyword occurrences (n = 44 keywords, 1080 total keywords identified).

The co-occurrence network of keywords (Figure 2) was generated using VOSviewer version 1.6.18. Analyzing the keywords from the retrieved articles, along with their visual representation as a co-occurrence network, provides valuable insights. From 85 scientific publications indexed in Scopus, a total of 1080 keywords were identified. These were sorted and categorized based on their frequency of occurrence in relation to PNS and oxidative stress–related diseases. This keyword clustering analysis was limited to the Scopus database, selected for its extensive coverage of life sciences and agricultural research, standardized metadata, and compatibility with VOSviewer.

We acknowledge that reliance on a single database may not fully capture the entire scope of PNS research, as relevant studies may also be indexed in PubMed, Web of Science, or regional databases. A complementary multi-database bibliometric analysis would provide a more comprehensive overview and could reveal additional research themes or geographic patterns. Nevertheless, Scopus remains one of the largest abstract and citation databases of peer-reviewed literature, and the clusters identified are consistent with the principal themes emerging from our broader literature review across multiple sources.

Figure 2 presents the network of terms that appeared at least five times, resulting in 44 keywords grouped into three clusters. The first cluster, represented in red, is primarily focused on bioactive compounds in the nut and its shell. It includes terms such as phenolic compounds, extraction, pecan nutshell, pecan nut, and plant extract. This cluster highlights research on extraction methods and the diverse bioactive compounds in PNS, aimed at elucidating their potential influence on human health. The second cluster, shown in green, relates mainly to in vivo and in vitro studies involving PNS and/or its extracts, including animal models and cell lines. Common terms in this group include animals, polyphenol, rats, and controlled experiments. This cluster illustrates the emphasis placed by researchers on linking PNS to health-promoting effects, particularly through oxidative stress modulation. The third cluster, depicted in blue and the smallest of the three, emphasizes human studies. It contains terms such as humans, cardiovascular diseases, cholesterol, and diet, reflecting a limited but emerging interest in the clinical and dietary implications of PNS. The relatively small size of the human studies cluster (blue) compared to the extraction/bioactive compounds (red) and preclinical study (green) clusters quantitatively confirm a significant translational gap: while chemical characterization and laboratory studies dominate the literature, clinical validation remains scarce. This visualization underscores the need for well-designed human intervention trials to translate promising preclinical findings into evidence-based therapeutic applications.

### 3.2. Pecan Nutshell as a Source of Phenolic Compounds

PNS contains diverse phytochemicals and bioactive compounds with significant biological potential. Recent studies have focused on understanding how plant-derived bioactive compounds exert their functions, with phenolic compounds emerging as a major area of interest. Nuts, including pecans, are well known to contain various phytochemicals, with phenolic compounds being the most prominent group [24,28]. Pecan shells have been shown to possess phenolic and flavonoid concentrations that are 5–20 times higher than those of the edible kernel [29]. Several phenolic acids have been identified, including gallic acid, vanillic acid, and caffeic acid in measurable amounts [29]. Pinheiro do Prado et al. [30] reported gallic acid concentrations ranging from 125–829 µg/mL, depending on the extraction type. Another study confirmed the presence of gallic acid and noted that pecan shells are also rich in fatty acids (monounsaturated, polyunsaturated, and saturated) [28]. Their analyses identified sterols, tocopherols, and many phenolic compounds, including gallic acid, ellagic acid, protocatechuic acid, p-hydroxybenzoic acid, and catechin, all of which demonstrate natural antioxidant activity [28]. More recently, Karuna et al. highlighted the presence of phenolic acids (gallic, caffeic, vanillic, ellagic, *p*-hydroxybenzoic), flavonoids (such as epigallocatechin), and lignin degradation products (such as lignols) in pecan shells [31].

To obtain these compounds efficiently while preserving their antioxidant activity, extraction parameters must be carefully optimized. Several critical factors determine both the yield and bioactivity of phenolic compounds from PNS. Temperature control plays a crucial role, as elevated temperatures (>80 °C) can enhance extraction efficiency by increasing solvent penetration and compound solubility, but excessive heat (>120 °C) may degrade thermolabile phenolics such as catechins and proanthocyanidins. Studies show that subcritical water extraction at 80 °C yields optimal ferric-reducing antioxidant power (FRAP) while minimizing degradation, whereas temperatures above 140 °C significantly reduce antioxidant capacity despite higher total phenolic yields [28,32]. Solvent selection and polarity are equally important, with aqueous ethanol (50–80% *v*/*v*) generally providing the best balance for extracting both hydrolyzable tannins (ellagic acid derivatives) and condensed tannins (proanthocyanidins), with 70% ethanol frequently reported as optimal. Pure water extracts primarily hydrophilic compounds but with lower overall yields, while pure organic solvents may extract lipophilic components but miss water-soluble phenolics [33,34]. Extraction time and method must also be considered, as prolonged extraction (>4 h) increases yields but may also extract undesirable compounds (e.g., oxidized phenolics, bitter tannins). Modern techniques like ultrasound-assisted extraction (UAE) and microwave-assisted extraction (MAE) reduce extraction time to 15–60 min while maintaining or improving antioxidant activity by minimizing thermal exposure and oxidative degradation [15]. pH and oxidative protection are critical since phenolic compounds, particularly catechols and galloyls, are susceptible to auto-oxidation at neutral to alkaline pH. Maintaining slightly acidic conditions (pH 3–5) during extraction and minimizing oxygen exposure through inert atmosphere or antioxidant addition (e.g., ascorbic acid), helps preserve antioxidant capacity. Shell preparation also significantly affects extraction efficiency, as particle size matters; finer grinding (≤0.5 mm) increases surface area and extraction rates but may also facilitate oxidation. Immediate extraction after grinding or storage under cool, dark, oxygen-free conditions is critical to prevent phenolic degradation. In summary, the most critical steps to maintain antioxidant activity during PNS extraction are: (a) temperature optimization (typically 60–80 °C for conventional methods), (b) appropriate solvent polarity (usually aqueous ethanol), (c) minimizing extraction time while maximizing efficiency (achieved through UAE/MAE), and (d) protecting against oxidative degradation through pH control and oxygen exclusion. These parameters collectively determine whether PNS extracts retain their therapeutic potential or yield degraded, low-activity products.

To obtain these compounds efficiently, extraction methods play a critical role. The type of solvent and extraction approach strongly determines the yield and efficacy of phytochemicals in pecan shells [32]. Conventional techniques, such as aqueous and alcoholic extractions (ethanolic or methanolic), remain widely used due to their simplicity and low cost [28,33]. Among these, aqueous ethanol extracts generally give the highest yields, while distilled water is less effective [34]. However, unconventional extraction methods, which include subcritical and supercritical fluids, sonication, microwave heating, infusion, stirring, and ball milling with deep eutectic solvents, have also been employed to enhance the recovery of bioactive compounds [27,34]. A study investigated subcritical water extraction, sonication-assisted extraction, and microwave heating, and found significant differences in total phenolic content (TPC) depending on extraction method, temperature, and by-product stream composition [32].

**Figure 3:**
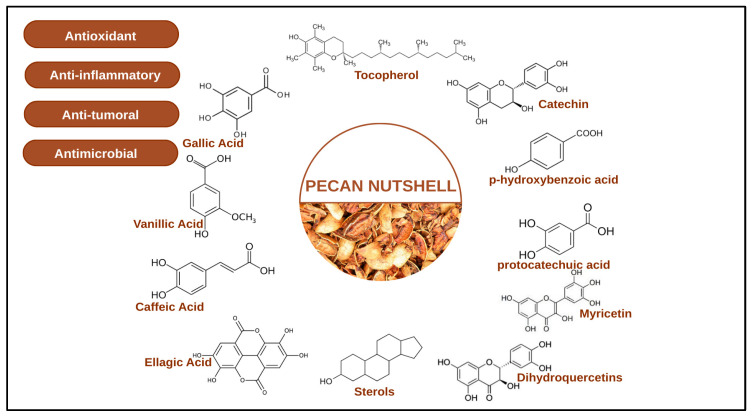
The various active compounds of pecan shell against oxidative stress. Major phenolic classes include hydrolyzable tannins (ellagic acid, gallic acid), condensed tannins (proanthocyanidins), and flavan-3-ols (catechin, epicatechin). These compounds neutralize reactive oxygen species (ROS) through multiple mechanisms including direct radical scavenging, metal chelation, and enhancement of endogenous antioxidant defenses.

The antioxidant properties of PNS are further influenced by external factors beyond the extraction process, including the pecan cultivar, growing environment, agricultural practices, and even the year of harvest [30]. These phenolic compounds exert their antioxidant activity primarily through neutralizing reactive oxygen species and protecting against oxidative stress, as illustrated in Figure 3.

The high concentration of phenols in pecan shells underlies their strong antioxidant potential. Moreover, extracts from pecan shells have demonstrated antimicrobial activity against Gram-positive bacteria, Gram-negative bacteria, and yeast microorganisms [35]. Animal studies reinforce these findings: dietary pecans reduced aberrant crypt foci in chemically treated male rats, suggesting a protective effect against early colon cancer through reduced oxidative stress [36]. Similarly, pecan shell extract protected Wistar rats against cyclophosphamide-induced oxidative stress [36]. Taken together, the evidence indicates that pecan shells are a rich reservoir of phytochemicals, particularly phenolic acids and flavonoids. Table 1 presents the major groups of phenolic compounds identified in PNS.

#### 3.2.1. Antioxidant Capacity of Pecan Nutshell

The PNS exhibits significant antioxidant capacity primarily due to its high content of phenolic compounds and flavonoids. PNS extracts exhibited antioxidant activity across multiple assays, including DPPH (2,2-diphenyl-1-picrylhydrazyl radical scavenging), ABTS (2,2′-azino-bis(3-ethylbenzothiazoline-6-sulfonic acid) radical cation decolorization), and FRAP (Ferric Reducing Antioxidant Power), with phenolic contents reported between about 116 to over 300 mg gallic acid equivalents (GAE) per gram of dry extract depending on extraction method and pecan variety. These phenolic components support their application in nutraceutical, food, and cosmetic industries. Pecan shells showed significantly higher antioxidant capacity (AC)—about 4.5 times greater than the kernels. A correlation (r^2^ = 0.61) was observed between total phenolic content (TPC) and AC-DPPH in the nutshells [39]. A previous study revealed significant differences in FRAP values for each extract. The highest AC was recorded for the extract obtained using the Accelerated Solvent Extraction (ASE) method at 100 °C. Temperature, byproduct type, and their interaction had significant effects (*p* < 0.01) on FRAP values. The ferric reducing capacity of PNS extract is largely attributed to the abundance of ellagic acid and gallic acid derivatives [28]. The highest activity was observed in extracts produced using the ASE method at 80 °C. Temperature, byproduct type, and their interaction had significant effects (*p* < 0.01). However, the correlation between ABTS values and TPC was relatively weak (r^2^ = 0.425) [28].

#### 3.2.2. Antioxidant Mechanisms of Action of Pecan Shell

PNS is a dense matrix of ellagic-acid derivatives, catechins and high-polymer proanthocyanidins that exert antioxidant defense thought multiple mechanisms:

1. Direct free-radical quenching: Ethanolic or aqueous PNS extracts from 20 cultivars show DPPH· and ABTS·^+^ inhibition up to ~3600 µmol Trolox g^−1^, values that track linearly with total phenolic content (150–490 mg GAE g^−1^) and confirm a primary hydrogen-atom/electron-donating capacity [33].
2. Metal chelation: Sub-critical-water extracts obtained at 80 °C display the highest ferric-reducing antioxidant power (FRAP) among industrial shell streams, reflecting multiple ortho-dihydroxyl and galloyl sites able to sequester Fe^3+^/Cu^2+^ and curb Fenton chemistry [28].
3. Recycling of endogenous defenses: In vivo, 5% (*w*/*v*) PNS aqueous extract given ad libitum prevents cyclophosphamide-induced spikes in testicular lipid peroxidation while restoring catalase and glutathione levels and normalizing superoxide-dismutase activity, indicating up-regulation or preservation of the cellular enzymatic antioxidant network [40].
4. Possible redox-signalling modulation: High-molecular proanthocyanidins in hydro-alcoholic PNS fractions have been docked in silico to Keap1 cysteine pockets; although direct Nrf2 read-outs are pending, the pattern suggests other tannin-rich botanicals known to trigger phase-II genes. Collectively, these converging mechanisms—radical scavenging, metal sequestration, enzyme rebalancing and putative Nrf2 activation—explain why PNS extracts consistently protect biological membranes, DNA and proteins in diverse oxidative paradigms while maintaining a favorable safety margin (rodent NOAEL ≥ 1 g/kg/day) [36]. Harnessing these actions through green extraction and tannin-enrichment technologies points to pecan shell as an inexpensive, circular-economy source of antioxidant ingredients for food, cosmetic and possibly therapeutic applications.

Given these multifaceted antioxidant mechanisms, it is essential to explore how pecan shell extracts may counteract oxidative-stress-driven pathologies such as endothelial dysfunction, a central feature of cardiovascular disease.

### 3.3. Therapeutic Potential of Pecan Nutshell Extracts Against Endothelial Dysfunction

Oxidative stress, which leads to disruption in endothelial function, plays an important role in the initiation and advancement of cardiovascular diseases. In the context of cardiovascular diseases, endothelial cells and vascular smooth muscle cells generate excessive reactive oxygen species (ROS), including superoxide anions [41,42]. Excess ROS overwhelm endogenous antioxidant defenses, leading to oxidative stress within the vascular wall. The produced ROS, especially superoxide ions, rapidly interact with nitric oxide (NO), an essential vasodilator synthesized by endothelial nitric oxide synthase (eNOS), consequently forming peroxynitrite. This interaction diminishes the bioavailability of NO, thereby restricting endothelium-dependent vasodilation and fostering vasoconstriction, inflammation, and thrombosis. Oxidative stress can induce eNOS uncoupling, wherein eNOS generates superoxide instead of NO, thereby further intensifying ROS production and exacerbating endothelial dysfunction [43]. This oxidative stress also activates inflammatory pathways, elevating adhesion molecule expression (ICAM-1, VCAM-1) and promoting leukocyte infiltration, atherosclerosis progression, and mitochondrial dysfunction collectively impairing vascular tone regulation and contributing to cardiovascular disease (CVD) pathogenesis [44,45,46,47].

Based on a comprehensive review of scientific literature, the therapeutic potential of PNS against endothelial dysfunction remains a promising but significantly underdeveloped field of research. While the shell is known to be a rich source of phenolic compounds with high antioxidant capacity confirmed in chemical assays (FRAP, DPPH and ABTS), direct evidence of its effects on human endothelial cells is absent. Furthermore, there are no clinical trials in humans evaluating the impact of pecan shell on cardiovascular health or endothelial function. However, there are some studies using in vivo rodent models to demonstrate its vascular effects which demonstrate fraction-dependent activity. Table 2 summarizes the available preclinical evidence, highlighting extract type, dosage, and vascular outcomes in different models. A condensed-tannin–enriched fraction (70% proanthocyanidins) proved most effective, showing a capacity to normalize elevated plasma levels of adhesion molecules ICAM-1 and VCAM-1 (by 27% and 24%, respectively) and restore thoracic aorta eNOS protein expression to 92% of control levels in mice following cigarette smoke withdrawal, at a dose of 50 mg/kg/day [48]. An aqueous total-phenolic extract demonstrated a different benefit, significantly reducing vascular permeability by 38% and tumor-associated micro-vessel density in a mouse cancer model, suggesting a stabilizing effect on the endothelial barrier [49]. In contrast, whole-shell powder, administered as a dietary supplement at doses up to 10 g/kg/day in rats, produced no observable changes in vascular histology or blood pressure [50].

The mechanism appears linked to antioxidant and anti-inflammatory actions, as evidenced by reduced lipid peroxidation and restored catalase activity in cardiac tissue in other rodent models [15]. The safety profile appears favorable, with high No-Observed-Adverse-Effect Levels (NOAELs) established in rats at ≥2 g/kg/day for the tannin fraction and 10 g/kg/day for the whole-shell powder, with no reported mutagenicity or significant organ toxicity [50,51]. In conclusion, while pecan shell extracts, particularly tannin-rich fractions, show potential in animal models for mitigating inflammation-driven endothelial dysfunction, the complete lack of human cell and clinical data represents a critical knowledge gap that prevents any firm conclusions on therapeutic efficacy.

The lack of vascular effects observed with whole-shell powder despite high doses (up to 10 g/kg/day) likely reflects bioavailability limitations rather than absence of bioactive compounds. Whole-shell powder contains approximately 55% insoluble fiber and only 3% phenolics, with the majority of antioxidant compounds likely bound within the lignocellulosic matrix [50]. Unlike purified extracts where phenolic compounds are released and concentrated through solvent extraction, whole powder phenolics remain largely inaccessible to intestinal absorption due to: (1) physical entrapment within fiber matrices, (2) strong tannin-protein-fiber interactions that resist gastric and intestinal digestion, and (3) limited surface area for enzymatic or microbial release in the gastrointestinal tract. Additionally, high-molecular-weight proanthocyanidins (>3000 Da), which constitute a major fraction in unprocessed shells, exhibit poor intestinal permeability. These findings underscore the critical importance of extraction and bioavailability enhancement strategies (e.g., enzymatic pre-treatment, nanoencapsulation, or fermentation) to translate PNS antioxidant potential into systemic vascular benefits. The contrasting efficacy of tannin-enriched fractions versus whole powder suggests that pharmaceutical-grade extraction is essential for therapeutic applications, while whole powder may serve better as a dietary fiber source with localized gastrointestinal antioxidant effects.

### 3.4. Potential of Pecan Nutshell Against Hyperglycemia

Hyperglycemia, or elevated blood glucose levels, leads to oxidative stress through the generation of excess reactive oxygen species (ROS) via multiple interconnected mechanisms. Excess glucose overloads the mitochondrial electron transport chain, stimulating enzymatic pathways (polyol, hexosamine, protein kinase C) that further increase ROS production, while also facilitating the formation of advanced glycation end products (AGEs), which in turn trigger inflammatory responses. This oxidative stress compromises insulin signalling and pancreatic β-cell functionality, perpetuating a cycle of hyperglycemia, ROS production, and metabolic disruption making oxidative stress a central contributor to diabetic complications [52,53,54,55]. PNS are abundant in phenolic compounds that possess antioxidant properties with potential antidiabetic effects through multiple mechanisms including digestive enzyme inhibition, β-cell protection, and metabolic pathway modulation.

In experimental animal studies, PNS have demonstrated protective characteristics against oxidative damage and some influence on glucose metabolism, although the outcomes differ based on the specific compound and its dosage [56]. Recently, research on PNS has moved from descriptive phytochemistry to mechanistic antidiabetic evaluation. The use of rodent model provided first-level translational support: daily gavage of an aqueous pecan shell extract (PSAE, 100 mg/kg for 28 days) in streptozotocin-diabetic Wistar rats lowered fasting glucose by ~28%, normalized triglycerides and total cholesterol, and attenuated disease-related weight loss, while genotoxicity (comet, micronucleus) remained negative—establishing a provisional NOAEL at the tested dose [55]. The same extract countered tyloxapol-induced hyperlipidaemia, hinting at hepatic lipid-metabolism modulation that could synergize with glycaemic control. Proposed mechanisms include digestive-enzyme blockade, ROS scavenging that protects β-cells, and putative AMPK activation, although the latter awaits molecular confirmation. However, no human trials or regulatory approvals for PNS-based antidiabetic nutraceuticals have been reported to date, leaving pharmacokinetics, chronic safety, and clinical efficacy as key knowledge gaps. The absence of dose–response studies, long-term safety data (>3 months), and lack of comparison with standard antidiabetic agents (metformin, sulfonylureas) in head-to-head trials further limits translation to clinical practice.

### 3.5. Potential of Pecan Nutshell Against Neurodegenerative Disorders

Given the established antioxidant mechanisms of PNS phenolics described in Section 3.2, and the central role of oxidative stress in neurodegeneration, this section evaluates both the general antioxidant capacity and specific neuroprotective evidence for PNS compounds. Neurodegenerative disorders including Alzheimer’s disease, Parkinson’s disease, amyotrophic lateral sclerosis (ALS), and Huntington’s disease are characterized by progressive neuronal loss, synaptic dysfunction, protein aggregation, and mitochondrial impairment, with oxidative stress serving as a common pathogenic thread [57,58,59]. While most neuroprotection studies of PNS have focused on antioxidant mechanisms, emerging evidence suggests additional protective pathways including cholinesterase inhibition, metal chelation in neural contexts, and potential anti-amyloid effects. However, the evidence base remains predominantly preclinical, with significant gaps in disease-specific animal models and human trials.

#### 3.5.1. Antioxidant Activity in Neurological Contexts

Beyond the general antioxidant mechanisms described in Section 3.2.2 (radical scavenging, metal chelation, endogenous defense enhancement, Nrf2 modulation), PNS phenolics have demonstrated specific relevance to central nervous system (CNS) protection through several neurologically relevant pathways.

The brain is particularly vulnerable to oxidative damage due to high oxygen consumption (20% of total body oxygen despite representing only 2% of body weight), abundant polyunsaturated fatty acids susceptible to lipid peroxidation, relatively low antioxidant enzyme concentrations compared to peripheral tissues, and high iron content that catalyzes Fenton reactions [60,61]. In a mouse model of withdrawal-induced oxidative stress, daily administration of PNS aqueous extract (30 mg/kg for 14 days) reduced hippocampal lipid peroxidation, restored catalase and superoxide dismutase (SOD) activity, and normalized anxiety-like behavior in the elevated-plus-maze, suggesting functional rescue of redox-sensitive limbic circuits [48]. While this model does not replicate Alzheimer’s or Parkinson’s pathology, it demonstrates that PNS polyphenols can cross the blood–brain barrier and counteract CNS oxidative imbalance.

Neuronal mitochondria are both major ROS sources and critical targets in neurodegeneration. Mitochondrial dysfunction precipitates neuronal apoptosis through cytochrome c release, ATP depletion, and calcium dysregulation [62]. Although direct evidence of PNS effects on neuronal mitochondrial function is lacking, extrapolation from peripheral tissue studies (cardiac mitochondrial protection in cyclophosphamide models [15]) suggests potential mechanisms. Future studies should employ isolated brain mitochondria or neuronal cell cultures (SH-SY5Y, primary cortical neurons) with mitochondrial function assays (oxygen consumption rate, membrane potential, complex activity) to validate CNS-specific mitochondrial protection.

Chronic neuroinflammation, mediated by activated microglia and astrocytes producing pro-inflammatory cytokines (TNF-α, IL-1β, IL-6) and ROS, drives progressive neuronal loss in multiple neurodegenerative diseases [63]. While no PNS studies have directly measured CNS inflammatory markers, the systemic anti-inflammatory effects observed in cardiovascular models (reduced ICAM-1/VCAM-1 [48]) suggest potential for neuroinflammation modulation. BV-2 microglial cells or primary astrocytes stimulated with lipopolysaccharide (LPS) are common models to test PNS effects on cytokine secretion, NF-κB activation, and microglial polarization (M1 vs. M2 phenotype).

#### 3.5.2. Neuroprotective Mechanisms Beyond Antioxidant Activity

While oxidative stress reduction remains the primary documented mechanism, emerging evidence suggests PNS may exert neuroprotection through additional, complementary pathways.

Acetylcholinesterase (AChE) and butyrylcholinesterase (BChE) inhibition is a validated therapeutic strategy for Alzheimer’s disease, as these enzymes degrade acetylcholine, a neurotransmitter critical for memory and cognition. Current drugs (donepezil, rivastigmine, galantamine) provide symptomatic relief by preserving cholinergic function. An in silico docking and in vitro enzymatic study found that low-molecular-weight proanthocyanidins from PNS form π-π stacking and hydrogen-bond networks within cholinesterase active sites, explaining their stronger binding affinity compared to larger tannin congeners. Specific IC_50_ values and kinetic parameters (competitive, non-competitive, or mixed inhibition) were not reported in the available literature but warrant investigation. No ex vivo validation using brain tissue homogenates or in vivo confirmation using cholinesterase activity in transgenic Alzheimer’s models (APP/PS1, 5xFAD mice) has been published. Behavioral validation through Morris water maze or Y-maze testing in cognitively impaired rodents is essential to confirm functional cognitive benefits.

Dysregulated metal homeostasis, particularly iron, copper, and zinc accumulation in brain regions affected by neurodegeneration, catalyzes ROS production and promotes protein aggregation (β-amyloid, α-synuclein, tau). The ortho-dihydroxyl groups in PNS catechins and galloyl moieties chelate these redox-active metals (demonstrated by FRAP assays [28]), potentially reducing metal-catalyzed oxidative damage and protein misfolding. Studies should test PNS phenolics in metal-overload neurodegeneration models (iron-induced substantia nigra damage for Parkinson’s; copper/zinc effects on amyloid aggregation kinetics using thioflavin-T assays) [64].

A review article reported that over 30 PNS-derived phenolics possess structural features associated with anti-amyloid activity, including aromatic rings that disrupt β-sheet stacking and hydroxyl groups that interfere with hydrogen bonding networks stabilizing amyloid fibrils [13]. However, no published studies have directly tested PNS extracts or purified PNS phenolics against amyloid-β aggregation using thioflavin-T fluorescence, transmission electron microscopy, or cell viability assays with neuronal cells exposed to amyloid oligomers. No studies in transgenic Alzheimer’s models (APP/PS1 mice) have examined brain amyloid plaque burden (immunohistochemistry, Aβ_40_/_42_ ELISA) after PNS treatment.

Therapeutic efficacy requires CNS bioavailability. The same review suggested PNS phenolics may cross the BBB based on structural predictions (molecular weight <500 Da for ellagic acid, catechins; lipophilicity parameters) [16]. The anxiety reduction observed in the mouse withdrawal study [48] provides indirect functional evidence of CNS penetration. Direct BBB permeability studies using: (a) Transwell systems with brain endothelial cells (bEnd.3, hCMEC/D3) measuring permeability coefficients (Papp); (b) In vivo pharmacokinetic studies with HPLC-MS/MS quantification of PNS phenolics in brain tissue, cerebrospinal fluid, and plasma after oral or intravenous administration; (c) microdialysis in awake, freely moving rodents to measure real-time CNS phenolic concentrations.

No studies have examined PNS effects on tau hyperphosphorylation (a hallmark of Alzheimer’s and other tauopathies) or α-synuclein aggregation/phosphorylation (central to Parkinson’s disease and Lewy body dementia). Cell-based assays (tau-GFP reporter cells, α-synuclein-transfected SH-SY5Y cells) and immunoblotting for phospho-tau (AT8, PHF-1 antibodies) or phospho-α-synuclein (Ser129) could establish whether PNS affects these critical pathological proteins.

In summary, PNS represent a polyphenol-rich, low-cost, sustainable source with plausible neuroprotective potential based on: (1) established antioxidant capacity that could mitigate the oxidative stress central to neurodegeneration, (2) preliminary evidence of CNS penetration and behavioral effects in one rodent study [49], (3) predicted cholinesterase inhibition from in silico/in vitro work [64], and (4) structural features suggesting metal chelation and anti-amyloid potential [16]. However, the evidence base is critically insufficient to support therapeutic claims. The field has focused disproportionately on general antioxidant characterization while neglecting disease-specific validation. Most published “neuroprotective” claims rest on antioxidant activity measured in non-neural systems, with limited direct neural relevance. Until PNS extracts are rigorously tested in established neurodegenerative disease models with appropriate behavioral, molecular, and histological endpoints, their neuroprotective potential remains speculative. The research priorities outlined above provide a roadmap for translating preliminary observations into evidence-based neuroprotective interventions.

### 3.6. Potential of Pecan Nutshell Against Cancer

Cancer is characterized by dysregulated cell proliferation, evasion of apoptosis, sustained angiogenesis, and metastatic potential [65]. Oxidative stress plays a paradoxical dual role in cancer: chronic ROS accumulation promotes carcinogenesis through DNA damage, mutagenesis, and activation of pro-survival signaling, while acute ROS elevation can trigger apoptosis in tumor cells [66,67]. Phenolic compounds identified in PNS, particularly ellagic acid, gallic acid, and proanthocyanidins, have demonstrated antiproliferative, pro-apoptotic, and anti-angiogenic effects in preclinical models [56,68,69,70,71]. However, the evidence base is heavily weighted toward in vitro cell line studies, with limited in vivo validation and no human clinical trials. This section organizes the available evidence by mechanism of action, prioritizing animal model data where available, and identifies critical gaps preventing clinical translation.

Only two in vivo anticancer studies exist. Female Swiss mice bearing Ehrlich ascites tumor (EAT) treated with crude aqueous PNS extract (100–200 mg/kg/day orally for 21 days) showed 52% tumor volume reduction, 67% survival increase, and apoptosis induction (confirmed by increased Bax/decreased Bcl-XL ratio) [50]. The same extract reduced tumor-associated micro vessel density by 31% in EAT-bearing mice, suggesting anti-angiogenic effects [49]. While these findings provide proof-of-concept for oral bioavailability and anticancer activity, the EAT model is a non-orthotopic, aggressive tumor system with limited relevance to human solid tumors. Critical gaps include: absence of orthotopic models (mammary fat pad, cecal implantation), no genetic cancer models (ApcMin/+, MMTV-PyMT), no metastasis models, no combination therapy studies with standard chemotherapeutics (doxorubicin, cisplatin), and no pharmacokinetic validation measuring tumor tissue concentrations of PNS phenolics. Consequently, it remains unknown whether IC_50_ values observed in vitro (15–138 µg/mL) are pharmacologically achievable in tumors after oral dosing.

In vitro studies demonstrate consistent cytotoxicity across multiple cancer cell lines through several mechanisms. (1) Apoptosis induction: MCF-7 breast cancer cells (IC_50_ = 74 µg/mL), MDA-MB-231 breast cancer (IC_50_ = 26 µg/mL for phenolic-enriched extract), HT-29 colon cancer (IC_50_ = 50–138 µg/mL with cultivar-dependent variability), and a panel including A549 lung, HeLa cervical, PC-3 prostate, and SK-MEL melanoma cells (IC_50_ = 15–60 µg/mL) all showed dose-dependent viability loss with apoptotic features (Annexin-V/PI positivity, caspase-3 activation, DNA fragmentation, Bax ↑/Bcl-XL ↓) [35,50,72,73]. (2) Cell cycle arrest: Phenolic-enriched extract (PCEE) induced G2/M phase arrest in MDA-MB-231 cells, though upstream regulators (cyclins, CDKs, checkpoint kinases) were not examined [35]. (3) Selective toxicity: PNS extracts showed minimal cytotoxicity to non-tumorigenic Vero cells while killing cancer cells, suggesting preferential targeting [73]. (4) Anti-angiogenesis: Beyond the in vivo microvessel density reduction noted above, the mechanistic basis (VEGF inhibition, VEGFR2 modulation) remains uninvestigated. (5) Oxidative stress modulation: PCEE paradoxically reduced ROS in doxorubicin-stressed CHO-K1 cells (cytoprotective) while presumably inducing ROS in cancer cells (cytotoxic), a concentration-dependent phenomenon requiring direct ROS measurement and NAC rescue experiments for validation [35]. Notably, all studies used complex extracts; bioassay-guided fractionation to identify active compounds (ellagic acid, gallic acid, specific proanthocyanidin oligomers) has not been performed, nor have synergistic combinations been characterized.

In summary, PNS extracts show promising in vitro anticancer activity (IC_50_ 15–138 µg/mL across six cancer types) and limited in vivo proof-of-concept (EAT tumor growth inhibition, survival benefit) with favorable preclinical safety (rodent NOAEL ≥ 2 g/kg) [50,51]. However, this evidence falls far short of supporting clinical recommendations. The complete absence of human trials, lack of pharmacokinetic data confirming tumor exposure, failure to identify active compounds, and over-reliance on cell culture without rigorous animal validation represent critical barriers to translation. Priority research recommendations include: (1) Immediate (1–2 years): Orthotopic breast cancer model (4T1 in Balb/c mice) with dose–response, survival, and metastasis endpoints; pharmacokinetic studies measuring plasma and tumor tissue concentrations of PNS phenolics after oral dosing; (2) Short-term (2–3 years): Bioassay-guided fractionation to identify active compounds; combination studies with doxorubicin or cisplatin to assess synergy; colon cancer chemoprevention in ApcMin/+ mice; (3) Long-term (3–5 years): If preclinical results are compelling, conduct GLP toxicology packages (90-day subchronic toxicity, genotoxicity, reproductive toxicity) for regulatory submission, followed by Phase I dose-escalation safety trial in cancer patients. The field has prematurely dispersed efforts across multiple cancer cell lines without achieving depth in any single cancer type; a focused strategy validating one cancer indication thoroughly (e.g., breast) would accelerate clinical translation more effectively than continued broad but shallow screening.

Purified phenolic compound–enriched extract (PCEE), derived from crude PNS extract, was evaluated for its antiproliferative and cytotoxic properties in MDA-MB-231 human breast cancer cells and CHO-K1 Chinese hamster ovary cells [35]. Treatment with PCEE for 48 h induced a concentration-dependent inhibition of MDA-MB-231 cell proliferation at concentrations of 25, 50, 100, and 200 mg/L. Morphological analysis revealed hallmark features of cell death following PCEE exposure, including progressive cellular shrinkage, membrane irregularities, cell rounding, reduced cell size, and loss of intercellular connectivity.

Mechanistically, PCEE exerted significant cytotoxic effects on MDA-MB-231 cells through induction of G2/M phase cell cycle arrest, thereby inhibiting tumor cell proliferation. Complementary evidence from Flores-Estrada et al. (2020) [34] demonstrated that extracts obtained from western and Wichita PNS exhibited dose-dependent antiproliferative effects on HeLa cervical carcinoma, A549 lung carcinoma, and LS180 colon adenocarcinoma cell lines, with the highest concentration (200 µg/mL) producing the most pronounced inhibition of cell growth. A comprehensive summary of these preclinical findings is provided in Table 3.

## 4. Perspective & Limitations

### 4.1. Perspective & Future Direction

PNS are emerging from an agro-industrial waste into a high-value source of polyphenols, lignans and melanin pigments with broad antioxidant potential. Over the past decade four converging trends have shaped this field, though significant gaps remain before clinical translation:

1. Green extraction has become mainstream: ball-milling combined with deep-eutectic solvents or water/ethanol systems now recover > 160 mg gallic-acid-equivalents g^−1^ dry shell while eliminating petrochemical solvents [74]. However, standardization across laboratories remains inconsistent, with extraction temperature, time, solvent-to-solid ratio, and particle size varying widely between studies, complicating reproducibility and comparison of bioactivity data.
2. PNS extracts consistently exhibit nanomolar-to-micromolar EC_50_ values in DPPH and ABTS assays and retain their antioxidant activity after incorporation into complex matrices, such as electrospun PAN fibers for skin-care applications, where radical scavenging reaches 60–80% and is accompanied by antimicrobial effects [20]. However, this strong in vitro antioxidant performance does not necessarily translate to in vivo efficacy, as evidenced by the absence of vascular effects following administration of whole-shell powder despite its antioxidant content (Section 3.3), underscoring bioavailability as a critical limiting factor.
3. First bio-efficacy signals have moved beyond in vitro chemistry: phenolic-rich water extracts trigger apoptosis in HT-29 colon-cancer cells (IC_50_ ≈ 50 µg/mL) [72], while lignin-rich fractions protect edible oils and margarines from oxidative rancidity as effectively as BHT. However, as detailed in Section 3.6, only one cancer type (breast, via EAT model) has in vivo validation, and no disease-specific models (transgenic Alzheimer’s, 6-OHDA Parkinson’s, orthotopic tumors) have been tested. The gap between in vitro promise and in vivo/clinical reality remains substantial.
4. Material-science approaches (silver-nanoparticle hybrids, zein microcapsules) are creating multi-functional carriers that couple antioxidant, antibacterial and controlled-release properties, broadening the application palette to foods, cosmetics and biomedical devices. These delivery systems may address the poor oral bioavailability of high-molecular-weight tannins, though comparative pharmacokinetic studies (nano encapsulated vs. free extracts) measuring plasma and tissue concentrations are not yet published.

To accelerate translation from agricultural waste to therapeutic resource, a systematic research pathway is proposed. This proposed research prioritizes: (1) Extraction optimization and standardization with quality control metrics (HPLC fingerprinting, marker compound quantification); (2) Comprehensive in vitro screening in disease-relevant cell models (primary neurons for neurodegeneration, patient-derived tumor cells for cancer) with mechanistic validation (Western blotting, gene expression, functional assays); (3) Disease-specific in vivo validation in appropriate animal models (transgenic, orthotopic, chemically induced) with pharmacokinetic profiling to establish dose-exposure-response relationships; (4) Safety assessment through GLP toxicology studies (acute, subchronic, genotoxicity, reproductive toxicity) to establish No-Observed-Adverse-Effect Levels (NOAEL) and identify potential side effects; (5) Formulation development to enhance bioavailability (nanoencapsulation, complexation, adjuvant co-administration); (6) Early-phase clinical trials starting with Phase I safety/tolerability studies in healthy volunteers, followed by Phase II proof-of-concept in target patient populations with biomarker endpoints. At each decision point, strict go/no-go criteria based on efficacy thresholds, safety margins, and pharmacokinetic feasibility should determine progression to the next stage. This approach contrasts with the current scattered landscape of isolated studies and would enable evidence-based prioritization of the most promising therapeutic applications.

### 4.2. Current Limitations and Research Gaps

Although existing research highlights the antioxidant and therapeutic potential of PNS, still translation into oxidative-stress-related disease management has not yet been achieved. Some of the key points related these gaps are:

Limited clinical evidence: Most studies on PNS are based on in vitro or animal models. Human clinical trials are scarce, limiting the generalization of findings to real-world settings. Currently, there are no known clinical trials that have systematically investigated the clinical or pharmacokinetic effect of PNS extract in human subjects. This absence is obvious taking into consideration several in vitro and in vivo studies that have established the anticancer properties and other health benefits that are derivable from extracts of PNS, including the demonstrable potent antioxidant properties, cytotoxic activity against oncogenic cells, induction of apoptosis and cell cycle arrest which are largely due to the phenolic compounds in this extract. The absence of clinical trials could be due to the lack of a comprehensive toxicological profile of the extracts obtained from PNS. However, the aqueous extract is considered safe for humans at moderate doses which could facilitate the initiation of clinical trials involving human subjects. Moreover, PNS extracts contain diverse types of phenolic compounds such as ellagic acid, gallic acid, protocatechuic acid and p-hydroxybenzoic acid which may interact to form complex mixtures with potentially synergistic effects, thereby complicating the undertaking of clinical trial studies due to the absence of data on the metabolic and bioavailability profiles of each of these components extracted from PNS [35,52].

Variability in extraction methods and composition: The extraction methods and solvents used in obtaining PNS extracts vary across studies, resulting in differences in phytochemical composition and bioactivity. Accordingly, extraction protocols should be rigorously standardized to guarantee reproducible levels of active constituents and batch-to-batch consistency, which are essential prerequisites for the feasibility of clinical trials. This lack of standardization makes it challenging to compare results and establish consistent therapeutic outcomes. Specific standardization needs include: (a) uniform particle size specifications (e.g., ≤0.5 mm mesh), (b) fixed extraction parameters (temperature, time, solvent-to-solid ratio), (c) validated analytical methods for marker compound quantification (HPLC with authenticated reference standards), and (d) stability testing protocols for extract storage.

Pharmacokinetics & bioavailability: Polyphenols in PNS may have limited bioavailability due to poor absorption, rapid metabolism, and excretion. Hence, PNS polyphenols are predominantly high-molecular-weight pro-anthocyanidins and lignols; their intestinal absorption, microbial biotransformation and systemic half-life remain unexplored. Strategies to improve their stability and bioavailability, such as nano-formulations or co-administration with other compounds, should be explored. Germ-free and antibiotic-depleted rodent models could clarify whether gut-derived metabolites (e.g., urolithins, valerolactones) mediate downstream redox signaling. Additionally, food matrix effects (co-consumption with fats, proteins, fiber) and inter-individual variability in gut microbiota composition may significantly influence PNS phenolic metabolism and bioavailability, necessitating both controlled pharmacokinetic studies and real-world effectiveness studies accounting for dietary context.

While preclinical safety studies have established favorable No-Observed-Adverse-Effect Levels (NOAEL ≥ 2 g/kg/day in rats) [50,51] with no reported mutagenicity or organ toxicity, several potential side effects warrant consideration before human use:

Condensed tannins represent a major fraction of PNS phenolics and are largely responsible for their biological activity. However, at high doses, tannins may exert unintended effects. They can precipitate dietary proteins, reduce the absorption of essential nutrients, particularly iron and calcium, induce astringency, and potentially irritate the gastrointestinal mucosa [75]. For this reason, daily intake should remain below levels associated with gastrointestinal discomfort (generally < 500 mg tannins/day in humans). Co-administration with vitamin C may help counteract potential reductions in iron absorption, and consumption with meals rather than on an empty stomach may attenuate astringency and improve tolerability [76]. For sensitive populations, the use of selectively extracted, tannin-reduced fractions could represent a safer alternative.

Tannins also exhibit non-specific protein-binding properties that may interfere with the absorption of concomitantly administered medications [77]. In addition, certain phenolic compounds can inhibit cytochrome P450 enzymes (such as CYP3A4 and CYP2D6) and drug transporters like P-glycoprotein, thereby potentially altering drug pharmacokinetics. To minimize such risks, a temporal separation of 2–3 h between PNS intake and critical medications is advisable [78]. Prior to human trials, in vitro drug–interaction studies (e.g., CYP inhibition and transporter assays) should be conducted. Early-phase clinical studies should exclude individuals receiving drugs with a narrow therapeutic index, such as warfarin, digoxin, or immunosuppressants, or include careful therapeutic drug monitoring [79,80].

Allergenicity must also be considered. Individuals with tree nut allergies may experience cross-reactivity to residual pecan shell proteins or shell-associated allergens (e.g., *Carya i* 1 and *Carya i* 2) [79,80]. Although shells generally contain lower protein levels than kernels, trace allergenic proteins may persist in aqueous extracts. Accordingly, extracts intended for human use should be tested for residual protein content (e.g., Bradford assay, SDS-PAGE), and individuals with known pecan or tree nut allergies should be excluded from early clinical trials. The development of highly purified, protein-free phenolic fractions may further reduce allergenic risk, and skin prick testing could be considered in sensitization assessments.

Another important consideration is the dose-dependent redox behaviour of phenolic compounds. While they act as antioxidants at physiological concentrations, supraphysiological doses may promote auto-oxidation, generating quinones and reactive oxygen species and thereby exerting pro-oxidant effects [81,82]. Establishing a maximum safe dose through carefully monitored Phase I dose-escalation studies is therefore essential, with concurrent evaluation of oxidative stress biomarkers such as 8-isoprostanes, malondialdehyde, and protein carbonyls. A consistent, moderate intake strategy is likely safer and more physiologically relevant than sporadic high-dose supplementation.

As agricultural by-products, pecan shells may also accumulate environmental contaminants, including heavy metals (lead, cadmium, arsenic, mercury) or pesticide residues [83]. Rigorous sourcing from low-contamination or certified organic regions, coupled with batch-specific ICP-MS analysis to verify compliance with regulatory standards (e.g., FDA, European Pharmacopoeia), is essential. Implementation of Good Agricultural and Collection Practices (GACP) would further strengthen quality control.

Finally, many plant phenolics exhibit hormetic effects, meaning that biological responses may be beneficial at low-to-moderate doses but ineffective or even harmful at very low or very high exposures [84]. PNS extracts may therefore possess a relatively narrow therapeutic window. Comprehensive dose–response studies in Phase I and II trials are required to define the optimal efficacy range. Importantly, clinical dosing strategies should avoid extrapolating “more is better” assumptions from in vitro findings and instead consider individualized approaches based on body weight, disease status, and inter-individual differences in metabolism, including gut microbiota–dependent phenolic biotransformation.

## 5. Conclusions

Pecan nutshells (PNS), long regarded as an agro-industrial waste, are increasingly recognized as a valuable and sustainable reservoir of bioactive compounds, particularly polyphenols such as gallic acid, ellagic acid derivatives, catechins, and proanthocyanidins. The evidence synthesized in this review demonstrates that PNS possess substantial antioxidant capacity, consistently outperforming edible kernels in total phenolic content and radical-scavenging activity. Through multiple converging mechanisms—including direct free-radical neutralization, metal chelation, modulation of endogenous antioxidant enzymes, and potential redox-signalling regulation—PNS extracts exhibit biologically relevant activity in preclinical models.

Across cardiovascular, metabolic, neurodegenerative, and oncological contexts, experimental findings suggest that PNS may mitigate oxidative stress–driven pathological processes. In animal models, tannin-enriched fractions have shown vascular protective effects, aqueous extracts have improved glycaemic parameters in diabetic rodents, and phenolic-rich fractions have demonstrated antiproliferative and pro-apoptotic effects in several cancer cell lines, with limited but promising in vivo confirmation. Moreover, preliminary evidence indicates possible central nervous system penetration and neurobehavioral modulation, although disease-specific validation remains insufficient.

Despite these encouraging data, the translational gap remains substantial. The current body of evidence is predominantly derived from in vitro studies and short-term animal experiments, with a complete absence of well-designed human clinical trials. Major barriers to clinical development include variability in extraction protocols, lack of standardized phytochemical characterization, limited pharmacokinetic and bioavailability data, and incomplete toxicological profiling for long-term use. In addition, the complexity of PNS polyphenolic mixtures, potential dose-dependent pro-oxidant effects, drug–polyphenol interactions, and inter-individual differences in gut microbiota metabolism must be systematically addressed before therapeutic recommendations can be formulated.

From a broader perspective, PNS exemplify the principles of circular economy and sustainable valorization of agricultural by-products. Their development as functional ingredients or adjunct therapeutic agents could simultaneously address environmental sustainability and public health challenges. However, advancement from promising bioactivity to clinically validated application requires a coordinated and methodologically rigorous research framework. Future priorities should include: (i) extraction standardization with batch-to-batch reproducibility; (ii) bioassay-guided fractionation to identify active constituents; (iii) comprehensive pharmacokinetic and metabolomic profiling; (iv) disease-specific validation in robust animal models; and (v) phased clinical trials assessing safety, tolerability, and biomarker-driven efficacy endpoints.

In conclusion, PNS have moved beyond the stage of preliminary antioxidant characterization and now stand at a pivotal point in translational development. While current evidence supports their potential as multifunctional antioxidant agents, definitive conclusions regarding clinical efficacy cannot yet be drawn. With systematic standardization, mechanistic clarification, and human validation, pecan nutshells may evolve from an underutilized agricultural by-product into a scientifically grounded, sustainable resource for the prevention or adjunctive management of oxidative stress–related disorders.

## Figures and Tables

**Table 1:** Major Phenolic Compounds Identified in Pecan Nutshells.

Compound Class	Specific Compounds Identified	Quantification Method	References
Hydrolysable Tannins	Ellagic acid derivatives, Ellagitannins, Gallic acid	Detected as prominent compounds via HPLC-ESI-MS/MS. Absolute μg/g quantification for individual compounds is not yet reported in the searched literature; their presence is confirmed by chromatographic peak areas and contributes to the total phenolic content (TPC) measured in GAE.	[37,38]
Condensed Tannins (Proanthocyanidins)	Proanthocyanidin dimers (A- and B-type), Trimer of (Epi)catechin–(Epi)catechin–(Epi)gallocatechin	Confirmed as a major fraction, with a reported concentration of 189 mg CE/g in an optimized extract. The presence of oligomers underscores the high degree of polymerization.	[38]
Flavones and flavonols	Myricetin, Dihydroquercetins	Identified as part of the broader flavonoid profile. Total flavonoid content has been quantified at 90 mg CE/g.	[38]
Flavan-3-ols	Catechin, Epicatechin, Gallocatechin	Serve as the monomeric building blocks for the abundant condensed tannins. Targeted quantification via MRM on LC-MS is feasible, with typical LOD/LOQ in the sub-µg/mL range.	[38]

**Table 2:** Preclinical Evidence for Pecan Nutshell Extracts on endothelial function and vascular health: Table shows stratification by model type (all in vivo animal studies; no in vitro or human available). Extract composition significantly influences bioactivity: purified tannin fractions show vascular benefits, while whole-shell powder shows no effects despite 100-fold higher doses, likely due to bioavailability limitations. Abbreviations: ICAM-1, intercellular adhesion molecule-1; VCAM-1, vascular cell adhesion molecule-1; eNOS, endothelial nitric oxide synthase; NOAEL, no-observed-adverse-effect level; TBARS, thiobarbituric acid reactive substances (lipid peroxidation marker); OECD, Organisation for Economic Co-operation and Development.

Extract/Fraction	Experimental Model	Dose	Observed Effect	Study Quality	References
Condensed-Tannin–Enriched Fraction (70% proanthocyanidins)	Swiss mice in a cigarette smoke withdrawal model	50 mg/kg/day (oral)	Normalized elevated plasma ICAM-1 and VCAM-1 levels (decreased by 27% and 24% vs. smoke-withdrawn controls); Restored eNOS protein expression in the thoracic aorta to 92% of air-control levels.	Positive controls included; dose–response limited	[48]
Aqueous Total-Phenolic Extract	Balb/C mice with Ehrlich ascites tumor (EAT)	100–200 mg/kg/day (oral)	Reduced vascular permeability by 38% in the peritoneum; Decreased tumor-associated micro-vessel density by 31%.	Cancer model; not cardiovascular disease-specific	[49]
Whole-Shell Powder (55% insoluble fiber, 3% phenolics)	Sprague-Dawley rats in a 13-week dietary study	5%, 10%, and 15% in diet (approx. 3.3, 6.7, and 10 g/kg/day)	No treatment-related changes in the histology of the aorta or cardiac vessels; No effect on systolic blood pressure.	OECD-compliant toxicity study; well-controlled	[50]
Condensed-Tannin–Enriched Fraction	Wistar rats in a 28-day oral toxicity study (OECD 407)	300, 1000, and 2000 mg/kg/day (oral)	No changes in serum ICAM-1 or VCAM-1 at any dose. Established a NOAEL of ≥2 g/kg/day.	Regulatory toxicity study; comprehensive organ pathology	[51]
Aqueous Shell Extract	Wistar rats with cyclophosphamide (CP)-induced oxidative stress	400 mg/kg/day (oral) for 10 days	Increased heart catalase (CAT) activity by 32% vs. CP control; Significantly decreased cardiac TBARS (lipid peroxidation) and plasma protein carbonyls; Restored circulating vitamin-C levels.	Oxidative stress model; mechanistic markers measured	[15]

**Table 3:** Preclinical Evidence for Anticancer Activity of Pecan Nutshell.

Extract/Fraction	Cancer Model	Dose/Conc.	Observed Effect (vs. Control)	References
Subcritical-water extract (“Native”, “Pawnee”)	Cervical, lung, skin, breast, colon, prostate cell panels	IC_50_ 15–60 µg/mL	>70% viability loss in tumor cells; non-cytotoxic to Vero cells; some extracts > doxorubicin potency	[73]
SPE-C18 phenolic-enriched extract (PCEE)	MDA-MB-231 & CHO-K1 cells	IC_50_ 26 µg/mL (MDA); 56 µg/mL (CHO)	G2/M arrest; ↓ ROS in doxorubicin-stressed cells (cytoprotection)	[35]
Optimised alkaline aqueous extracts (“Mahan”, “Marameck)	HT-29 colon cancer cells	IC_50_ 50–138 µg/mL	Apoptosis confirmed by Annexin-V/PI; caspase-3 activation	[72]
Crude aqueous shell extract	MCF-7 cells & Ehrlich ascites tumor in mice	100 µg/mL (in vitro); 100–200 mg/kg/day (in vivo)	46% in vitro apoptosis; ↓ tumor volume; 67% ↑ survival; Bax ↑/Bcl-XL ↓	[50]

## Data Availability

No new data were created or analyzed in this study. Data sharing is not applicable to this article.
